# Feasibility trial of a scalable psychological intervention for women affected by urban adversity and gender-based violence in Nairobi

**DOI:** 10.1186/s12888-016-1117-x

**Published:** 2016-11-18

**Authors:** Katie S. Dawson, Alison Schafer, Dorothy Anjuri, Lincoln Ndogoni, Caroline Musyoki, Marit Sijbrandij, Mark van Ommeren, Richard A. Bryant

**Affiliations:** 1 University of New South Wales, Sydney, Australia; 2 World Vision International/Australia, Melbourne, Australia; 3 World Vision Kenya, Nairobi, Kenya; 4 Psychological Support Center, Nairobi, Kenya; 5 VU University Amsterdam, Amsterdam, Netherlands; 6 Department of Mental Health and Substance Abuse, World Health Organization, Geneva, Switzerland; 7 School of Psychology, University of New South Wales, Sydney, NSW 2052 Australia

**Keywords:** Gender based violence, Trauma, Mental health, Psychological intervention, Trial

## Abstract

**Background:**

Living in conditions of chronic adversity renders many women more vulnerable to experiencing gender-based violence (GBV). In addition to GBV’s physical and social consequences, the psychological effects can be pervasive. Access to evidence-based psychological interventions that seek to support the mental health of women affected by such adversity is rare in low- and middle-income countries.

**Methods:**

The current study evaluates a brief evidence-informed psychological intervention developed by the World Health Organization for adults impacted by adversity (Problem Management Plus; PM+). A feasibility randomised control trial (RCT) was conducted to inform a fully powered trial. Community health workers delivered the intervention to 70 women residing in three peri-urban settings in Nairobi, Kenya. Women, among whom 80% were survivors of GBV (*N* = 56), were randomised to receive five sessions of either PM+ (*n* = 35) by community health workers or enhanced treatment as usual (ETAU; *n* = 35).

**Results:**

PM+ was not associated with any adverse events. Although the study was not powered to identify effects and accordingly did not identify effects on the primary outcome measure of general psychological distress, women survivors of adversity, including GBV, who received PM+ displayed greater reductions in posttraumatic stress disorder symptoms following treatment than those receiving ETAU.

**Conclusions:**

This feasibility study suggests that PM+ delivered by lay health workers is an acceptable and safe intervention to reach women experiencing common mental disorders and be inclusive for those affected by GBV and can be studied in a RCT in this setting. The study sets the stage for a fully powered, definitive controlled trial to assess this potentially effective intervention.

**Trial registration:**

ACTRN12614001291673, 10/12/2014, retrospectively registered during the recruitment phase.

## Background

Common mental disorders contribute significantly to the global disease burden [1]. This is also true in low- and middle-income countries (LMICs), where exposure to adverse living conditions, such as living in slums and chronic poverty, is high. Residing in such settings makes people vulnerable to experiencing risk factors for common mental disorders, as well as gender-based violence (GBV).

According to a global review by the World Health Organization (WHO), 35% of women worldwide report having experienced physical and/or sexual intimate partner violence or non-partner sexual violence [2]. In 2014, among a representative sample of people in Nairobi Kenya, 54% and 20% of women, aged 15–49 years, reported having experienced physical violence and sexual violence, respectively, with the main perpetrators being intimate partners [3]. The rates of different forms of violence in slums within Nairobi can be expected to be higher as having a low-income increases the risk of violence among Kenyan women [3].

As one of the most prevalent human rights violations, the harmful effects of GBV are well established [4–7]. At an individual level, the effects extend across physical (e.g. physical injury), sexual (e.g. sexually transmitted diseases, pregnancy and chronic pelvic pain), and social (e.g. stigma and rejection by family and community, marital strain and increased victimization) domains. With respect to mental health, female survivors of GBV are at risk of developing depression, anxiety and posttraumatic stress disorder [4, 7]. They are more likely to be suicidal and abuse substances [4]. In addition, many women report suffering from low self-esteem and somatic complaints, engaging in deliberate self-harm and risky sexual behaviours, and social isolation [7]. Such mental health challenges undermine women’s functional capacity and can further position them to re-victimisation [8]. Finally, GBV is associated with economic and education consequences, negatively impacting productivity and development of societies and communities [4].

In most LMICs the mental health effects of adversity including GBV, as with common mental disorders generally, often go untreated. This situation exists primarily because of inadequate resources that are allocated to mental health needs [9, 10]. In response to this situation there has been a shift towards utilization of non-specialist providers in delivering scalable variants of evidence-based psychological interventions (i.e. ‘task-shifting’) [10]. There is growing evidence for the applicability of psychological interventions by non-specialists in LMICs [11, 12].

Few psychological interventions targeting survivors of GBV in LMIC have undergone rigorous scientific evaluation and therefore their efficacy and effectiveness remain indeterminate [5, 13]. One randomized controlled trial conducted by Bass and colleagues [14] investigated the effectiveness of group cognitive processing therapy (CPT), a trauma-focused psychological intervention. Compared to individual support alone (comprising psychosocial, legal, financial and medical support), group CPT plus individual support was much more effective in reducing symptoms of PTSD, depression and anxiety, for Congolese women affected by GBV. At 6-month follow-up, women who received group CPT plus individual support were also much less likely to have a diagnosis of probable PTSD, depression or anxiety. This study was conducted within long-standing, confidential programming for Congolese GBV survivors, which made it possible to study the impact of a trauma-focused intervention on a sample existing exclusively of GBV survivors.

In most communities of the world it is very difficult to provide care exclusively to GBV survivors, because of confidentiality issues involved in organizing such services. Such confidentiality issues are especially important in communities where the stigma of GBV may lead to further violence and possible abandonment. While there likely will always be a role for dedicated GBV services, there is an urgent need for integrating care of GBV-survivors within general health, social and mental programs that offer psychological help to people affected by GBV without requiring an explicit discussion on GBV.

This paper describes a feasibility trial for a brief psychological intervention aimed at alleviating symptoms of common mental disorders in women exposed to adversity. The intervention was delivered by community health workers (CHWs) to women in peri-urban villages in Nairobi, Kenya who were affected by all forms of adversity, including possible GBV.

## Methods

### Setting

This study occurred in three peri-urban villages that are part of the primary health care system of Dagoretti sub-county in Nairobi, Kenya. According to the World Bank data indicators, Kenya is a LMIC, with almost 46% of its population living under the poverty line [15]. Health services are expensive for many Kenyans, with 80% of people unable to access medical care coverage [16]. There is a scarcity of human resources working in the mental health sector, with approximately one psychiatrist per 500 000 [17].

The study was implemented by World Vision Kenya and was approved by the Ethics Review Board at the Great Lakes University of Kenya, Nairobi and the WHO Ethical Review Committee.

### Participants

The study was conducted from March to September 2014 in three low-income peri-urban regions of Nairobi. The program was offered to all women impaired by psychological distress. Potential participants were women recruited from community screening of 890 conducted by trained assessors. Inclusion criteria included: (a) female, (b) over 18 years of age, and (c) three or above on the General Health Questionnaire (GHQ-12; a measure of general anxiety and depression) [18] *and* 17 or above on WHO Disability Assessment Schedule- version 2.0 (WHO-DAS 2.0; a measure of functional disability) [19]. These cut-offs were used to ensure that only women with both marked distress and impairment were recruited.

Individuals were excluded from the study if they were considered to be at risk of ending their life or displayed severe mental disorder (i.e. psychotic disorders and substance dependence) or severe cognitive impairment (i.e. severe intellectual disability or dementia). Individuals meeting exclusion criteria were referred to a local hospital where mental health services were available. Women who reported a major traumatic event or death of a loved one within the last month were encouraged to wait one month after the event before completing screening. This was to allow for natural remission and not engage participants in treatment unnecessarily. Similarly, when a woman presented with a new, imminent protection risk (e.g. loss of shelter or recent GBV that posed an imminent risk to her safety) their physical safety was ensured before engaging them in the trial. In the latter two instances, women were re-contacted after a period of time to re-assess their wellbeing and interest in the study. The characteristics of participants are presented in Table 1.

**Table 1:** Participant characteristics

	PM+	ETAU
Age (mean, years)	33.32 ± 8.70	37.56 ± 10.46
Education (mean, years)	9.25 ± 4.34	8.91 ± 4.44
Married	50.0%	57.1%
Employed	67.9%	71.4%
Trauma History		
Disaster	47.8%	60.0%
Fire	43.5%	40.0%
Road accident	47.8%	44.4%
Serious accident	43.5%	30.0%
Chemical exposure	26.1%	20.0%
Physical assault	65.2%	63.2%
Assault by weapon	34.8%	26.3%
Sexual assault	26.0%	15.0%
Unwanted sexual experience	26.1%	10.0%
War exposure	13.0%	10.0%
Life-threatening illness	47.8%	65.0%
Witness violent death	26.1%	26.3%
Unexpected death of loved one	47.8%	55.0%
Total Number of Traumas (mean)	5.87 ± 3.42	5.70 ± 2.60
Suicidal Thoughts (last fortnight)	10.7%	9.5%

### Measures

Assessments were conducted at baseline and one to two weeks after the scheduled 5^th^ session of intervention (or approximately 6 weeks later for the control group). Instruments that were not available in Kiswahili were translated and back-translated specifically for this project. All measures were administered by assessors who received four-day training in data collection strategies and how to support an individual if they were showing signs of distress. All assessments were delivered in interview format to address variable levels of literacy in the sample.

### Psychological distress

The GHQ-12 was employed to index general psychological distress in women. The GHQ-12 comprises 12 questions scored on a 4-point Likert scale ranging from 0 to 3. When used as a screening tool, the GHQ-12 is typically scored bi-modally (i.e., 0-0-1-1), with scores ranging from 0 to 12. The GHQ-12 has been widely used across LMICs, including Kenya [20]. In our study, a cut-off of three or higher was applied to indicate clinical levels of psychological distress.

### Functioning

Functioning and disability was measured with the WHO-DAS 2.0. This is a 12-item instrument that asks people how much difficulty they have had completing activities covering six domains (cognition, mobility, self-care, getting along, life activities, and participation), due to their illness in the last 30 days. Difficulties are scored on a 5-point Likert scale (*none* to *extreme*). The WHO-DAS 2.0 has demonstrated validity as screening and outcome measures, displaying good sensitivity to change [21, 22], and has been validated in Kenya [23].

### Gender-based violence

Five key questions from the WHO Violence Against Women Instrument (WHO-VAW) [24], developed for use in the WHO Multi-Country Study on Women’s Health and Domestic Violence [25] and previously used in Kenya [3], were included in assessment. Women were asked to indicate the frequency of physical and sexual violence they had experienced by a partner or other adult since the age of 15. Given the sensitive nature of these questions, women were reminded that that their responses would be kept confidential and that they could decline answering questions any time.

### Stressful life events

To index exposure to stressful and/or traumatic life events, the Life Events Checklist [26] was utilised. The measure consists of a list of 17 experienced or witnessed events, such as rape, serious injury, or the sudden death of a loved one. A Kiswahili version of the assessment was applied [27]. At the post-treatment assessment the question phrasing was adapted so as to only capture events that had occurred since commencing the study.

### Post-traumatic stress symptoms

The PTSD Checklist for DSM-V - Civilian Version (PCL-5) [28] was used to measure symptoms of PTSD. This is a 20-item checklist corresponding with the 20 DSM-5 PTSD symptoms. Items are rated on a 5-point Likert scale (*not at all* to *extremely*) and add up to a total severity score of 80. The PCL-5 was adapted to ask for symptoms in the last week (rather than month) to enhance sensitivity to change. As the PCL-5 was not available in Kiswahili, the English version was translated and checked via back-translation.

### Treatment conditions

#### Problem management plus

PM+ is an innovative, evidence-informed and scalable intervention that aims to provide psychological support to adults exposed to adversity. Specifically, it aims to address common symptoms of mental disorders such as depression, anxiety and stress, and client self-identified practical problems, such as interpersonal conflict and financial problems. The pilot version of PM+ that was tested involved five, 90-min sessions, that combined evidence-based strategies of problem solving counselling with selected behavioural strategies including stress management, behavioural activation, in vivo exposure and strengthening social support. [29]

PM+ underwent a comprehensive phase of adaptation to the culture and context of Kenya. Adaptations were informed by ethnographic study, translation of the materials, community consultations and review of the content and final blind back translation of key elements. Adaptations were documented using the Bernal and Saez-Santiago framework [30]. Based on these processes, relevant changes were made to the PM+ training manual, the PM+ manual for lay counsellors, and the supporting worksheets for clients.

The training and supervision of CHWs sought to uphold a task-shifting approach [31]. This process of delegation involves moving the primary delivery of healthcare from specialists such as psychiatrists and psychologists to less specialised workers in order to maximise the efficient use of health workforce resources. In this study, the intervention providers were women engaged in community health work with the government.

In Kenya CHWs have varied levels of education and do not receive any training or experience in mental health care (encompassing counselling, psychology or psychiatry). An eight-day training program was delivered by the master trainer (KSD), directly to the CHWs (*N* = 23) and three Kenyan psychologists who would provide supervision to the CHWs. Training included the provision of basic theoretical knowledge of common mental disorders, basic counselling skills, delivery of the PM+ intervention and self-care practices. The classroom training was followed by four weeks of practice cases (approximately three clients per CHW) under close supervision. CHWs were then required to pass competency assessments before offering PM+ to participants involved in the feasibility study. CHWs also received training in psychological first aid in order to know how to react in case people were exposed to new traumatic events during the study [32].

The CHWs were supervised on a weekly basis by one of three local supervisors who were clinical psychologists with previous experience in providing clinical supervision. The local supervisors were supervised weekly to fortnightly for one to two hours by the master trainer and a fourth local supervisor (LN). Supervision comprised of building skills in the PM+ intervention as well as in training and supervision of CHWs with emphasis on research principles, such as standardisation and fidelity of treatment. Thus, supervision was cascaded from a foreign intervention specialist to local experts, and onwards to CHWs.

### Enhanced treatment as usual (ETAU)

ETAU consisted of receiving care from primary care clinicians (nurses) at one of three local primary healthcare clinics (PHCs). For the purposes of this study and given that treatment as usual for mental disorders in this setting often equates to no care, primary care nurses – who had already training and experience in counselling people with HIV/AIDS – received one-day training in psychological first aid and an additional day of training in supportive counselling, based on the International Federation of Red Cross manual [32], without follow-up supervision.

### Treatment fidelity

Attempts were made to audio record 15% of the sessions, however, all but two participants refused consent due to concerns about confidentiality. Instead, CHWs and nurses documented the treatment components they delivered for each session. In the PM+ condition, this was checked against the specific components to be delivered according the manual. These forms were reviewed in supervision and in an attempt to maintain fidelity to the treatment, supervisors identified when fidelity was breached and CHWs were required to deliver the treatment component in the subsequent session. Project staff met periodically with nurses providing the ETAU to establish the types of support and counselling they offered to participants in this condition and check that there was no contamination of PM+ strategies being used in the ETAU condition.

### Procedure

Screening was conducted in the community by trained independent assessors. Assessors were assigned to CHW catchment areas and approached every tenth household in their allocated area. After selecting a household, assessors first determined how many female household members over 18 years lived there and randomly selected one woman to invite for confidential screening. If a woman over the age of 18 years refused participation, assessors continued invite additional female members of the household, or moved on to the next household. All participants completed informed consent for screening. Participants who screened positive were invited into the study, andcompleted informed consent for pre-assessment and treatment allocation. Pre-assessment was expected to occur within the following two weeks, however due to a number of implementation challenges this was significantly delayed. Challenges included difficulties contacting or locating clients for the assessment, clients’ lack of understanding about the project and expectations of receiving material support, family members refusing to give permission for client’s to participate, and clients lack of trust in receiving treatment from CHWs. Fourteen participants declined treatment after it was offered. In total, 200 women remained who could be randomized, however the pilot study only required 70 participants to achieve its goals of determining feasibility, and so 70 women were randomly selected for enrolment in the pilot study.

**Fig. 1:**
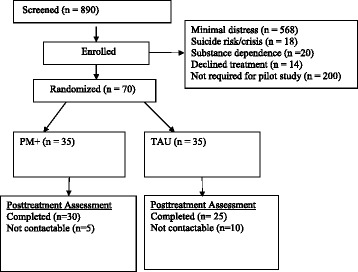
Participant flow

Following the assessment, included participants were informed that they would be randomly allocated to either PM+ or ETAU by an independent researcher. Randomization was performed using computerized software by an independent colleague (i.e. off-site in Sydney and not involved in the trial). Figure 1 summarises the participant flow. Seventy women were randomized into the study and allocated to either PM+ (*n* = 35) or ETAU (*n* = 35).

Post-treatment assessments were completed by independent assessors who were unaware of the treatment condition of participants. Blindness was maintained by ensuring that assessors who conducted assessments did not have access to (a) condition allocation of participants or (b) participant notes. In addition, careful attention was paid to ensure assessors had no contact with CHWs or ETAU nurses by having them work in different locations. Any adverse reactions reported spontaneously by the participant or observed by research team reported to a local independent advisory board, that comprised an independent medical officer, an independent counselling psychologist, the site principle investigator, and a clinical supervisor. An adverse reaction was defined as any undesirable experience occurring to a participant during the study, whether or not considered related to the research procedure.

Fifty-five participants (76%) completed the post-treatment assessment (30 from PM+ group and 25 from the ETAU group). No serious adverse events were reported during the course of treatment or at post-treatment assessment.

### Statistical analysis

Notably, as this was a feasibility trial that was intended to indicate acceptability, safety, and viability of the intervention in this population; it was not powered to detect significant differences. To index the relative effects of the two treatment conditions, univariate analyses of covariance (ANCOVAs) for each of the three sets of continuous measures (GHQ, WHO-DAS, PCL) using the pre-treatment scores as covariates were performed. We focus on completer analyses because this study is intended to be exploratory only. Treatment effect sizes were between treatment conditions at post-treatment. We derived Cohen’s *d* effect size by calculating the mean difference between assessments of each treatment condition and dividing this by the pooled standard deviation. We used Hedges’ *g* effect sizes to correct for variations due to small sample sizes.

## Results

Table 1 presents the participant characteristics, including trauma exposure. Of the 70 women included in the exploratory study, 56 (80%) reported experiences of GBV (29 in PM+ and 27 in ETAU). Women in both conditions were exposed to more than 5 traumatic events on average. Therefore, whilst all 70 participants may not have personally experienced GBV, they were part of a community with substantial adversity, trauma exposure and living in a community where GBV is rife. There were no differences detected between participants in the PM+ and ETAU conditions on any of the pre-treatment outcome measures, demographics, or trauma exposure.

Table 2 presents the mean values for GHQ, WHO-DAS, and PCL. A univariate ANCOVA of post-treatment GHQ scores controlling for baseline GHQ scores indicated a non-significant effect for treatment condition [*F*(1, 52) = 0.45, *p* = .51, ƞ = .01]. A univariate ANCOVA of post-treatment WHO-DAS scores controlling for baseline WHO-DAS scores indicated a non-significant effect for treatment condition [*F*(1, 46) = 0.88, *p* = .35, ƞ = .02]. A univariate ANCOVA of post-treatment PCL scores controlling for baseline PCL scores indicated a significant effect for treatment condition [*F*(1, 53) = 4.19, *p* = .04, ƞ = .07]. Specifically, women who received PM+ reported significantly lower PCL scores following the intervention than those who received TAU. The between group effect sizes at post-treatment for the GHQ, WHO-DAS and PCL were, 0.35, 0.35, and 0.50, respectively.

**Table 2:** Mean psychopathology scores

	PM+	ETAU
Pre-Treatment		
GHQ-12	17.66 ± 3.83	19.48 ± 5.08
WHO-DAS 2.0	22.69 ± 5.61	25.45 ± 6.78
PCL-5	27.86 ± 19.70	26.26 ± 18.42
Post-Treatment		
GHQ-12	10.57 ± 4.42	12.24 ± 5.515
WHO-DAS 2.0	14.87 ± 4.93	16.88 ± 6.19
PCL-5	9.29 ± 10.96	16.76 ± 17.71

*Note. GHQ-12* general health questionnaire-12, *WHO-DAS 2.0* WHO disability assessment scale- version 2, *PCL-5* PTSD checklist for DSM-5

## Discussion

This study aimed to test the feasibility and acceptability of a novel, scalable psychological intervention with Kenyan women exposed to a range of adverse life events. Although the study was not powered to detect clinically significant differences on outcome measures, the findings revealed that PM+ had the potential to improve mental health, particularly a reduction in PTSD symptoms for women affected by adversity, including GBV. The intervention did not have a significant effect on measures of general distress (GHQ) or functional impairment (WHO-DAS) compared with ETAU. This may be because the concepts measured by these instruments by comparison to the PCL are too heterogeneous to detect effects in a small sample size.

No adverse or serious adverse events were reported by women receiving the intervention, suggesting that PM+ did not cause harm or exacerbate distress beyond one’s capacity to cope with it. PM+ did not appear to result in increased risk to their safety and no instances of stigma associated with receiving the intervention was reported. This is a particularly important finding given a significant proportion of the sample reported GBV. Relatedly, this shows that a generic screening approach that does not directly target GBV survivors was still effective in capturing these women.

Finally, the successful implementation of PM+ in peri-urban communities with non-specialised health workers suggests that Kenyan CHWs can competently deliver PM+ after brief training and under regular supervision. This also speaks to the acceptability of PM+, suggesting it most likely met the necessary culturally-centered standards of this context. The rates of drop-out at post-assessment also provide some support to acceptability of PM+. Eighty-six percent of women who received the intervention were willing to participate in the post-assessment, compared to 71% of ETAU women. These findings highlight the need for the intervention to be validated with a fully powered RCT.

Implementation of this feasibility trial was met with a number of challenges associated with the research procedures and treatment that have required examination before embarking on subsequent large-scale trials. Firstly, review of the screening and pre-assessment procedures has been necessary to improve communication with eligible individuals and their family members about the role of the project to set appropriate expectations. Training assessors in managing questions about what might and might not be involved in the treatment may help to overcome these difficulties. Challenges associated with maintaining contact with eligible clients was a particular challenge with this community, with many women not having reliable access to phones. Secondly, women were often engaged in casual employment, meaning they were unable to provide consistent times when they would be at home. On many occasions assessors would attend their house at a pre-arranged time only to find the individual was not home because they were engaged in work that day.

Pertaining to the implementation of the treatment, PM+ handouts relied on clients being literate and CHWs feeling comfortable completing forms. In most instances these handouts were not utilised for these two reasons. Accordingly, a revised version of PM+ has changed the client handouts into pictorial representations of the PM+ strategies.

Secondly, it was revealed through supervision processes that the in vivo *exposure* strategy was rarely implemented and when it was, it was done so incorrectly. For instance, in vivo exposure was applied to women reporting symptoms of cognitive worry as opposed to anxious avoidance. In vivo exposure was also not used in an exploratory trial of PM+ in Peshawar, Pakistan [33]. In vivo exposure is arguably a more complex strategy to train non-specialist providers to deliver effectively, although other research groups have managed this [34]. In this study, in vivo exposure required the CHW to identify the source of anxious avoidance, develop a gradual exposure plan and to accompany the client with the initial steps of this plan. It is possible the specific source of avoidance was difficult for CHWs to define and that accompanying the client was logistically prohibitive; therefore in vivo exposure was readily abandoned as a strategy to implement with clients. Consequently, the revised version of PM+ omits in vivo exposure [35].

The grossly inadequate number of trained mental health professionals in LMICs remains a notable barrier to many people receiving adequate mental healthcare. This study adds to the growing evidence in support of task-shifting approaches in the delivery of evidence-informed psychological interventions [12, 36, 37]. More needs to be known about who are the best people to train to deliver such interventions. In this study, existing volunteer workers in the community were recruited. Their familiarity with the health system and the community was advantageous and their passion for improving the overall health of people in their community facilitated their engagement in the program. However, their main occupation in providing healthcare to the community often impacted on their availability and engagement in training and delivering sessions to clients. Moreover, CHWs reported concerns about an overwhelming increase in workload without adequate reimbursement. Throughout the course of the study, monetary incentives had to be negotiated to motivate CHWs to remain involved in the study. Also, there were a few instances of women feeling uncomfortable to engage in PM+ with their CHW due to the sensitive nature of the topics discussed. In Kenya, it is likely necessary to have further discussion regarding the employment - whether on a voluntary basis, with or without incentives, salaried or accompanied with alternative income generation initiatives - of various lay workers to implement interventions such as PM+, where the treatment remains free of charge to the client.

A related barrier to accessing mental healthcare is the capacity for interventions to be scaled-up into local government ministries or non-government organisations that often have limited budgets for mental health and psychosocial support. An integral component of PM+ is a robust training and supervision model. Our use of an outside master trainer for both training and supervision is unsustainable and may restrict local partners and staff from taking ownership of and fully accepting the intervention. As the support for evidence-based treatments delivered by non-specialist providers in LMICs grows, future research needs to give attention to training and supervision models that promote sustainability.

Limitations of the study design include the small sample size and conducting analyses on treatment completers. However, it should be emphasised that this was a pilot study that only allowed for pre- and post-assessment time points, precluding a more comprehensive evaluation of the intervention. We also note that the PCL-5 was specifically translated into Kiswahili for this study, and accordingly it lacks psychometric properties. The difficulties recruiting women to this pilot study will inform strategies for more effectively engaging participants in the subsequent larger controlled trial. Specifically, better education about the goals and content of the project was identified in the pilot trial as crucial to secure better recruitment. We also note that identifying the optimal level of psychological distress or impairment is challenging in LMIC settings, such as Nairobi, where there is a dearth of established norms for psychological well-being. The mean score on the GHQ-12 of women in this study (19.22) is somewhat lower than one previous study in Kenya of internally displaced persons (28.70) [38], which may be explained in part by the different sampling methods. It was not the intention of this study to determine the optimal method for identifying psychological impairment in Kenya but we recognize this is an important challenge for future community programs.

## Conclusions

The enduring mental health impact of urban adversity and GBV is a global health concern that has been the focus of substantial commentary in the last two decades. However, there is a scarcity of rigorous scientific study into the effectiveness of feasible psychological interventions addressing mental health problems in this population in LMICs. The current study provides a proof-of-concept for the feasibility of a brief psychological intervention in Kenyan women exposed to urban adversity, including GBV. As well as having the potential to alleviate symptoms of common mental disorders in this population, the findings suggest that PM+ can be delivered by lay health workers, is accepted in this setting, and that it is feasible to test the intervention in a RCT. A large-scale RCT is required to test the effectiveness of PM+ among GBV survivors in similar settings.

## Abbreviations

CHW: Community health worker
CPT: Cognitive processing therapy
ETAU: Enhanced treatment as usual
GBV: Gender-based violence
GHQ: General health questionnaire
LMIC: Low and middle income countries
PCL: PTSD checklist
PM+: Problem management plus
PTSD: Posttraumatic stress disorder
WHO: World Health Organization
WHO-DAS: World Health Organization Disability Assessment Schedule
